# Detection rates and factors affecting thereof in endometrial hyperplasia, endometrial carcinoma, and cervical glandular lesions on cervical smear

**DOI:** 10.1002/cam4.6376

**Published:** 2023-07-27

**Authors:** Joanna K. M. Ng, Bryan H. C. Cheung, Dennis H. Y. Lee, Joshua J. X. Li, Philip P. C. Ip, Jacqueline H. S. Lee, Carol S. Y. Yeung, Mei‐Yung Yu

**Affiliations:** ^1^ Department of Anatomical and Cellular Pathology Prince of Wales Hospital, The Chinese University of Hong Kong Sha Tin Hong Kong; ^2^ Department of Pathology Queen Mary Hospital, School of Clinical Medicine, The University of Hong Kong Pok Fu Lam Hong Kong; ^3^ Department of Obstetrics and Gynaecology Prince of Wales Hospital, The Chinese University of Hong Kong Sha Tin Hong Kong

**Keywords:** cervical adenocarcinoma, cervical smear, endometrial carcinoma, endometrial hyperplasia

## Abstract

**Introduction:**

Endometrial lesions are morphologically diverse and uncommon on cervical smears, with its detection rate and associated diagnostic categories uncharacterized. In this study, cervical smears matched to histologically proven endometrial hyperplasias and carcinomas were reviewed and compared with cervical in‐situ‐carcinomas/carcinomas, aiming to detail the diagnostic performance of cervical smears for upper tract and glandular lesions.

**Methods:**

Pathology reports of cervical smears, hysterectomies, endometrial and cervical biopsies from 1995 to 2021 were retrieved. Diagnoses of cervical smears were matched to endometrial hyperplasias and carcinomas, or cervical carcinomas and reviewed.

**Results:**

Totally 832 cervical smears (272 cervical carcinomas, 312 endometrial carcinomas, and 248 hyperplasias) were included. Considering all cytologic glandular diagnosis as positive, the detection rate of cervical adenocarcinoma‐in‐situ was the highest (64.3%), followed by cervical adenocarcinoma (63.8%), endometrial carcinoma (31.7%), and hyperplasia (with atypia–8.5%; without atypia–2.3%) (*p* < 0.001). Endometrial hyperplasia was most often diagnosed as atypical squamous cells of undetermined significance (ASCUS) (5.0%) or atypical glandular cells, not otherwise specified (3.6%) without indication of endometrial origin. For endometrial carcinomas, higher FIGO grading and endocervical involvement were associated with higher detection rates across all diagnostic categories (*p* = 0.002–0.028). High FIGO grade was associated with suspicious/favor neoplastic (C4) (31.1%vs10.3%, *p* < 0.001) and carcinoma (C5) (17.8% vs. 5.6%, *p* = 0.005) categories, but not for all glandular diagnoses combined (33.3% vs. 31.0%, *p* = 0.761).

**Conclusion:**

Detection rates for endometrial lesions are lower than cervical lesions but not insignificant. Endometrial hyperplasia should be recognized as a differential of human papilloma virus‐negative ASCUS and prompt consideration of investigation of the upper genital tract.

## Graphical abstract

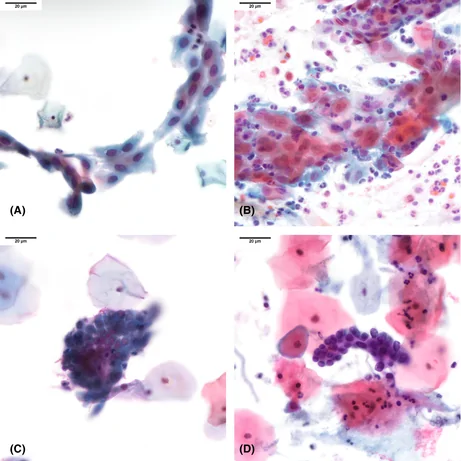

## 1 INTRODUCTION

Cervical smear is an effective screening and diagnostic method for cervical pathologies, and is robust for detection of cervical squamous lesions.^1^ However, endometrial lesions can be sampled inadvertently on cervical smears.^2^, ^3^ Owing to the broad morphological spectrum and constantly evolving classification systems of endometrial lesions^4^ and rarity in cervical smears, the detection rate and reporting of endometrial lesions, in particular endometrial hyperplasia, is not well understood. Currently there is no exclusive cytologic diagnostic category in the Bethesda system for reporting of cervical cytology for endometrial hyperplasia,^5^ and such cases variably presents with a cytologic diagnoses of benign endometrial cells,^6^ atypical glandular cells (AGC), or even squamous abnormalities.^7^ As cervical smears are performed in a large and often asymptomatic population,^8^ it can be expected that a significant number of patients with early asymptomatic endometrial neoplasms will have undergone cervical smear, including incidental screening. In addition, although cytomorphologic criteria have been described for diagnosis of endometrial cancers on pap smears, reproducibility is imperfect and also may not be applicable for endometrial hyperplasia.^5^ It is of interest whether cervical smears are capable of detecting endometrial neoplasms, and under which cytologic diagnostic category are they reported as benign endometrial cells.^9^

In this study, a large number of cervical smears matched to histologically proven endometrial hyperplasia and endometrial carcinoma were reviewed retrospectively and referenced smears of histologically confirmed cervical adenocarcinoma‐in‐situ, adenocarcinoma, and carcinoma, aiming to compare detection rates between different types of female genital tract lesions and to identify clinicopathological features of endometrial lesions associated with a positive cervical smear diagnosis. The distribution of positive cytologic diagnostic categories were also reviewed. Findings in this study will establish the performance of cervical smear in detection of human papilloma virus (HPV)‐negative endometrial lesions, which will be important for decisions on selection of HPV‐testing or retaining morphological assessment.^10^

## 2 METHODS

A computerized search of the department pathology archives of the Prince of Wales Hospital from the year 1995 to 2021 was performed. The pathology reports of cervical smears (including conventional and liquid based), biopsy (cervical punch, endometrial punch, aspiration, and curettage), and resection (cone excision, hysterectomy) specimens with the diagnoses of any cervical carcinoma, endometrial carcinoma, and endometrial hyperplasia were retrieved. Patient demographics, staging data and diagnosis were retrieved from the reports. All equivalent cytologic diagnoses were reclassified according to the Bethesda system for reporting of cervical cytology if possible,^5^ and descriptive or non‐specific diagnoses were categorized to the five‐tiered cytologic diagnoses (inadequate, benign/negative, atypia, suspicious for malignancy, and malignant). In cases where multiple concomitant diagnoses, diagnoses of glandular lesions were considered over squamous abnormalities. For all cases with hysterectomy performed, the hospital records of the respective patient were reviewed for complete disease staging (to include nodal status and presence of metastasis). These retrieved cases were matched with cervical smears up to 180 days prior to surgical specimens (hysterectomy, conization, or loop electrosurgical excision), or up to 7 days after and 180 prior to biopsy specimens by an identity number unique to each patient. For cases with multiple matched specimen, the highest‐grade histologic diagnosis was considered as the final diagnosis. Exclusion criteria were cases with a lesion that the site of primary could not be determined, cases with concurrent cervical and endometrial carcinomas and metastatic carcinomas.

Statistical analysis was performed using SPSS (version 23.0). The chi‐squared test was used to compare the diagnostic rate at binary cutoffs for different types of cervical and endometrial lesions, and histologic and staging parameters in endometrial carcinomas associated with detection on cervical smears. A *p* < 0.05 was considered significant. The study was approved by The Joint Chinese University of Hong Kong—New Territories East Cluster Clinical Research Ethics Committee with waiver of the requirement of written informed consent (2022.530).

## 3 RESULTS

A total of 832 cervical smears from 754 patients, with an average age of 52.1 years (23–92 years) were reviewed, which included 172 cases of non‐glandular cervical carcinoma (squamous cell carcinoma, and carcinoma, not specified), 42 cervical adenocarcinoma‐in‐situ and 58 cervical adenocarcinoma, 312 endometrial carcinoma, and 248 endometrial hyperplasia (Table 1). No cases of histologically proven squamous cell carcinoma reported concomitant squamous and glandular abnormalities (i.e., no squamous abnormality was omitted due to presence of glandular abnormalities).

**TABLE 1:** Cytological diagnoses of the cohort.

	Squamous abnormality only				Glandular abnormality or abnormality, NS							
	SCC	Other squamous abnormalities	Inadequate	Negative	AGC, NOS	Endometrial cells seen	Atypia, NS	AGC, favor neoplastic	Suspicious for malignancy, NS	Adenocarcinoma/AIS	Carcinoma, NS	Total
Cervix												
Adenocarcinoma‐in‐situ	0	7	2	6	16	0	0	3	0	8	0	42
Adenocarcinoma	0	8	3	10	8	0	0	11	2	15	1	58
Squamous cell carcinoma^a^	26	107	7	9	3	1	2	0	7	0	2	164
Carcinoma, NS	0	0	2	2	0	0	0	0	2	1	1	8
Endometrium												
Carcinosarcoma	0	1	0	2	0	0	0	0	0	0	0	3
Clear cell carcinoma	0	0	1	0	0	0	0	1	0	2	0	4
Endometrioid carcinoma												
Grade I^b^	0	8	12	95	32 (3)^c^	3	0	8	2	5	1	166
Grade II	0	6	10	29	11	0	2	1	0	7	0	66
Grade III	0	3	4	11	1	0	0	1	0	3	0	23
Not graded	0	0	1	7	1 (1)^c^	1	0	1 (1)^c^	1	1	1	14
Mixed carcinoma	0	0	0	3	0	0	0	1	0	0	0	4
Serous carcinoma	0	2	0	6	0	0	0	1	3	3	0	15
Carcinoma, NS	0	2	3	7	2	0	0	0	1	2	0	17
Endometrial hyperplasia												
With atypia	0	7	16	84	7	0	1	0	0	2	0	117
Without atypia	0	8	20	100	2	0	0	1	0	0	0	131
Total	26	159	81	371	83	5	5	29	18	49	6	832

Abbreviations: AGC, atypical glandular cell; AIS, adenocarcinoma‐in‐situ; NOS, not otherwise specified; NS, not specified; SCC, squamous cell carcinoma.

^a^ No cases of histologically proven SCC reported concomitant squamous and glandular abnormalities (i.e., no squamous abnormality was omitted due to presence of glandular abnormalities).

^b^ Includes two cases of mucinous carcinoma diagnosed under previous versions of the World Health Organization.

^c^ Bracketed are cases with diagnosis or comments suggesting endometrial origin.

Considering any diagnosis of glandular lesion or atypia as positive, the detection rate of cervical adenocarcinoma‐in‐situ was the highest (64.3%, *n* = 27/42), followed by cervical adenocarcinoma (63.8%, *n* = 37/58), endometrial carcinoma (31.7%, *n* = 99/312), and the lowest being endometrial hyperplasia (5.2%, *n* = 13/248) (*p* < 0.001). In comparison, there were 15 cases of endometrial hyperplasia diagnosed in the category of squamous abnormality on cervical smear (6.0%, *n* = 15/248). For cervical carcinomas (including adenocarcinoma‐in‐situ), the diagnosis rate for atypia (C3) (84.9% vs. 38.8%, *p* < 0.001) and carcinoma (C5) (19.9% vs. 8.0%, *p* < 0.001) were significantly higher than endometrial carcinomas. For reference, the detection rate for non‐glandular cervical carcinomas was 88.4% (*n* = 152/172).

Comparing all glandular lesions of the cervix and endometrium, the detection rate was consistently higher for cervical glandular lesions including (64.0% vs. 20.0%, *p* < 0.001) and excluding endometrial hyperplasia (64.0% vs. 31.7%, *p* < 0.001), and also at all cytologic diagnostic categories (at least suspicious/favor neoplastic [C4]—40.0% vs. 14.7%, *p* < 0.001; carcinoma [C5]—24.0% vs. 8.0%, *p* < 0.001) (Table 2). As for subgroup analysis, hyperplasia with atypia had a higher glandular atypia rate than hyperplasia without atypia (8.5% vs. 2.3%, *p* = 0.027). Endometrial carcinoma had higher detection rates across all cytologic categories (any glandular lesion/atypia [C3]—31.7% vs. 5.2%, *p* < 0.001, at least suspicious/favor neoplastic [C4]—14.7% vs. 1.2%, *p* < 0.001 and carcinoma [C5]—8.0% vs. 0.8%, *p* < 0.001) (Table 3).

**TABLE 2:** Comparison of diagnosis rate of cervical versus endometrial glandular lesions (a) including endometrial hyperplasia and (b) excluding endometrial hyperplasia

	Cervical	Endometrial	*p*‐Value
(a) Including endometrial hyperplasia			
Unsatisfactory/negative for glandular lesions (C1–C2)^a^	36	448	
Any glandular atypia or lesion (C3–C5)^b^	64	112	
	64.0%	20.0%	<0.001
(b) Excluding endometrial hyperplasia			
Unsatisfactory/negative for glandular lesions (C1–C2)^a^	36	213	
Any glandular atypia (C3–C5)^b^	64	99	
	64.0%	31.7%	<0.001
Unsatisfactory/negative/atypia (C1–C3)^a^	60	266	
At least suspicious (favor neoplastic) glandular lesion (C4–C5)	40	46	
	40.0%	14.7%	<0.001
Non‐malignant diagnosis (C1–C4)^a^	76	287	
Carcinoma (non‐squamous) (C5)	24	25	
	24.0%	8.0%	<0.001

^a^ Cytological diagnoses of squamous abnormalities only considered as negative (i.e., negative for glandular lesions).

^b^ Includes endometrial cells seen.

**TABLE 3:** Comparison of diagnosis rate between (a) endometrial hyperplasia with and without atypia, and (b) endometrial hyperplasia and carcinoma.

	Hyperplasia without atypia	Hyperplasia with atypia	*p*‐value
(a) Endometrial hyperplasia with and without atypia			
Unsatisfactory/negative for glandular lesions (C1–C2)^a^	128	107	
Any glandular atypia (C3–C5)^b^	3	10	
	2.3%	8.5%	0.027
Unsatisfactory/negative/atypia (C1–C3)^a^	130	115	
At least suspicious (favor neoplastic) glandular lesion (C4–C5)	1	2	
	0.8%	1.7%	0.463
Non‐malignant diagnosis (C1–C4)^a^	131	115	
Carcinoma (non‐squamous) (C5)	0	2	
	0.0%	1.7%	0.163
(b) Endometrial hyperplasia and carcinoma			
Unsatisfactory/negative for glandular lesions (C1–C2)^a^	235	213	
Any glandular atypia (C3–C5)^b^	13	99	
	5.2%	31.7%	< 0.001
Unsatisfactory/negative/atypia (C1–C3)^a^			
At least suspicious (favor neoplastic) glandular lesion (C4–C5)	245	266	
	3	46	
Non‐malignant diagnosis (C1–C4)^a^	1.2%	14.7%	< 0.001
Carcinoma (non‐squamous) (C5)			
	246	287	
	2	25	
	0.8%	8.0%	< 0.001

^a^ Cytological diagnoses of squamous abnormalities only considered as negative (i.e., negative for glandular lesion).

^b^ Includes endometrial cells seen.

For endometrial carcinomas, comparing high‐grade and low‐grade carcinomas, there was no difference in the rate of reporting any glandular lesions (33.0% vs. 31.0%, *p* = 0.761), but high‐grade endometrial carcinomas were more frequently reported as at least suspicious/favor neoplastic (C4) (31.1% vs. 10.3%, *p* < 0.001) and carcinoma (C5) (17.8% vs. 5.6%, *p* = 0.005) rates (Table 4). There were 293 cases of endometrial carcinoma matched with hysterectomy performed, consisting of 225, 29, 18, and 21 stage I, II, III, IV diseases, respectively (Table S1). A FIGO stage of II or above and presence of endocervical involvement were associated with higher rates in all cytologic categories of glandular lesions (*p* = 0.002–0.028). Regional nodal metastasis showed association only with the any glandular lesion category (*p* < 0.001) but not for cytologic diagnoses of higher grades. Myometrial invasion, advanced local disease, and presence of distant metastasis did not affect detection rates (*p* > 0.05) (Table 4).

**TABLE 4:** Factors affecting detection of endometrial carcinomas on pap smear.

	Total	Unsatisfactory/negative for glandular lesions (C1–C2)^a^	Any glandular atypia (C3–C5)^b^	At least suspicious (favor neoplastic) glandular lesion (C4–C5)	Carcinoma (non‐squamous) (C5)
Histological grade					
Low‐grade	232	160 (69.0%)	72 (31.0%)	24 (10.3%)	13 (5.6%)
High‐grade	45	30 (66.7%)	15 (33.3%)	14 (31.1%)	8 (17.8%)
*p*‐value			0.761	< 0.001	0.005
FIGO stage					
Stage I	225	166 (73.8%)	59 (26.2%)	24 (10.7%)	10 (4.4%)
Stage II‐IV	68	38 (55.9%)	30 (44.1%)	68 (100%)	8 (11.8%)
			0.005	0.015	0.028
Myometrial invasion					
Superficial (<50%)	183	129 (70.5%)	54 (29.5%)	25 (13.7%)	9 (4.9%)
Deep (≥50%)	104	70 (67.3%)	34 (32.7%)	13 (12.5%)	8 (7.7%)
*p*‐value			0.574	0.780	0.339
Endocervical involvement					
Present	46	23 (50.0%)	23 (50%)	12 (26.1%)	7 (15.2%)
Absent	247	181 (73.3%)	66 (26.7%)	34 (13.8%)	10 (4.0%)
*p*‐value			0.002	0.005	0.005
Advanced local disease (adnexal, parametrial, serosal, or vaginal involvement)					
Yes	18	13 (72.2%)	5 (27.8%)	3 (16.7%)	1 (5.6%)
No	275	191 (69.5%)	84 (30.5%)	36 (13.1%)	17 (6.2%)
*p*‐value			0.805	0.665	0.915
Regional nodal metastasis					
Present	9	1 (11.1%)	8 (88.9%)	6 (66.7%)	1 (11.1%)
Absent	284	203 (71.5%)	81 (28.5%)	36 (12.7%)	17 (6%)
*p*‐value			< 0.001	0.072	0.528
Distant metastasis					
Present	21	15 (71.4%)	6 (28.6%)	3 (14.3%)	2 (9.5%)
Absent	272	189 (69.5%)	83 (30.5%)	36 (13.2%)	16 (5.9%)
*p*‐value			0.852	0.891	0.503

^a^ Cytological diagnoses of squamous abnormalities only considered as negative (i.e., negative for glandular lesion).

^b^ Includes endometrial cells seen.

## 4 DISCUSSION

Non‐squamous and upper tract lesions are not uncommonly encountered in cervical smears,^2^, ^11^ these lesions are often problematic not only due to their rarity and relative thus reduced experience in part of the assessor, but also due to the broad differential diagnoses.^12^, ^13^ The incidence of endometrial carcinomas in asymptomatic populations subjected to screening cervical smears is low, reported to be at less than 2 per 1000 women years in prospective cohorts.^14^ As such, cohort studies are only able to identify a limited number of patients with endometrial pathologies,^15^ including those based on colposcopy units receiving referrals.^16^, ^17^ The majority of studies on the detection of upper tract lesions on cervical smears with sufficient statistical power are case‐controls studies.^18^ Diagnosis of endometrial hyperplasia is more difficult^19^ with lower incidence than invasive carcinomas.^20^ The current study, based on a large retrospective cohort, was able to determine the diagnostic performance of cervical smears on endometrial lesions, particularly in addressing the relatively low detection rate and non‐specific cytologic diagnosis of endometrial hyperplasia on cervical smears.

A PubMed literature search using the keywords “cervical smear”/“cervical cytology” and “endometrial hyperplasia” yielded only retrospective cohorts reviewing cases of AGC and capturing isolated cases of endometrial hyperplasia^16^, ^17^, ^21^, ^22^ (Table 5). An expanded search including all endometrial lesions yielded larger cohorts, but the number of cases with endometrial carcinomas heavily outnumber that of endometrial hyperplasias.^7^, ^23^ The case number of endometrial hyperplasia were limited,^7^, ^23^ with the exception of Milicić et al, yielding 126 cases of endometrial hyperplasia which necessitated a review of over 5000 smears^24^ (Table 5). In addition, a series of cervical smears with benign endometrial cells identified, limited to postmenopausal women only, endometrial hyperplasia was found in 16 out of 61 patients.^6^ With the transition to HPV testing over morphological evaluation in cervical screening programs, the possible advantage of detecting HPV‐negative endometrial lesions is important in the decision of retaining morphological assessment or not.^10^

**TABLE 5:** Reported cases of endometrial hyperplasia detected on cervical smear.

	Population	Results	Cytologic diagnoses
Current	832 smears with histologic follow‐up	248 endometrial hyperplasia (117 with atypia, 131 without atypia)	15 squamous abnormalities, 9 AGC, 1 AGC favor neoplastic, 2 adenocarcinoma/AIS, 1 atypia not specified
Al‐Rayyan et al.^16^	68 smears of AGC	7 endometrial hyperplasia	7 AGC
Sawangsang et al.^17^	63 smears of AGC	1 endometrial hyperplasia	1 AGC
Cakmak et al.^21^	73 smears of AGC	3 endometrial hyperplasia	3 AGC
Scheiden et al.^22^	191 smears of AGC	3 endometrial hyperplasia with atypia	3 AGC
Milicić et al.^24^	5105 smears with follow‐up	126 endometrial hyperplasia	7 abnormal diagnoses, including 2 specifying endometrial origin
van Doom et al.^7^	543 smears from patients with postmenopausal bleeding	6 endometrial hyperplasia with atypia	1 mild dyskaryosis
Kaur et al.^23^	134 smears from patients with postmenopausal bleeding	13 endometrial hyperplasia without atypia	No abnormal cytologic diagnosis
Wu et al.^6^	61 smears with benign endometrial cells identified from postmenopausal patients	11 endometrial hyperplasia with atypia, 5 endometrial hyperplasia with atypia	16 benign endometrial cells seen

Abbreviations: AGC, Atypical glandular cells; AIS, adenocarcinoma‐in‐situ.

In this study, cervical smears from patients with histologically proven endometrial carcinomas and endometrial hyperplasias, from a tertiary gynecologic oncology referral center serving a population of over 1 million, were reviewed. Cervical carcinomas, including adenocarcinoma‐in‐situ, adenocarcinoma, and squamous cell carcinoma with matched cervical smears from the same period were also retrieved for reference.

The detection rates, including cytologic diagnoses, of any glandular lesion were highest for cervical glandular lesions. Even for the subgroups of endometrial carcinoma with endocervical involvement and endometrial hyperplasia with atypia, detection rates for cervical glandular lesions were superior. Although the atypia (including squamous atypia) rate for cervical adenocarcinoma was lower than squamous cell carcinoma, a trend was observed for cervical adenocarcinomas over squamous cell carcinoma for a diagnosis of invasion. The detection rates of cervical glandular lesions were also consistently above those of endometrial lesions, including and excluding endometrial hyperplasia at all cytologic diagnosis categories (Table 4).

**FIGURE 1:**
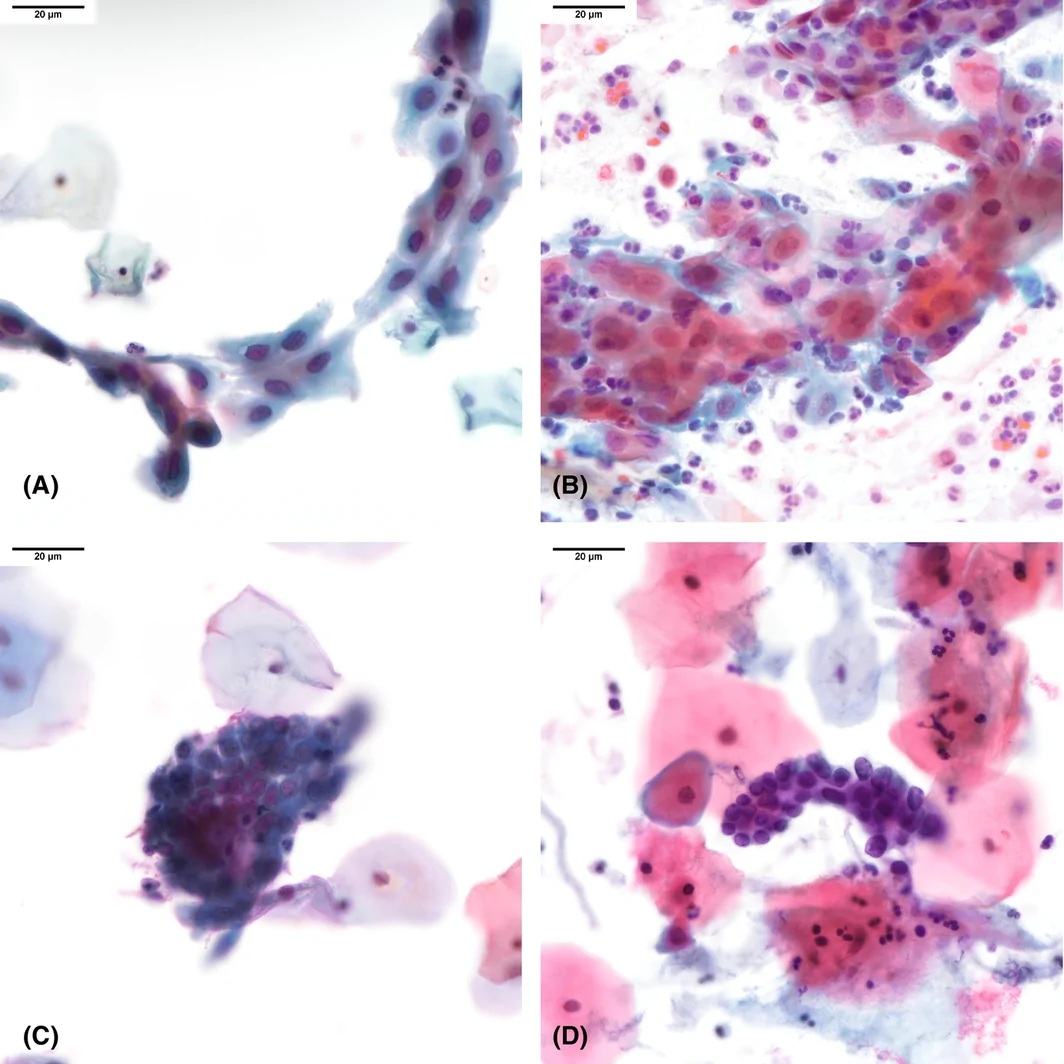
Cervical smears of endometrial hyperplasia. (A) Endometrial hyperplasia without atypia, cytologic diagnosis was atypical squamous cells of undetermined significance (ASCUS), 400× magnification; (B) Endometrial hyperplasia with atypia, cytologic diagnosis was ASCUS, 400× magnification; (C) Endometrial hyperplasia without atypia, cytologic diagnosis was atypical glandular cells, not otherwise specified (AGC, NOS), 400× magnification; (D) Endometrial hyperplasia with atypia, cytologic diagnosis was AGC, NOS, 400× magnification.

As for endometrial hyperplasia, hyperplasia with atypia showed a higher rate of atypical glandular lesion than hyperplasia without atypia. Endometrial hyperplasia was most frequently diagnosed as atypical squamous cells of undetermined significance (ASCUS) (*n* = 12) and atypical glandular cells, not otherwise specified (AGC, NOS) (*n* = 9) (Figure 1; Table 1). Only one case of hyperplasia without atypia was diagnosed as AGC, favor neoplastic, and two cases of hyperplasia with atypia was diagnosed as adenocarcinoma. Of the 12 cases of endometrial hyperplasia diagnosed as ASCUS, six were confirmed by hysterectomy without histologic evidence of squamous intraepithelial‐lesion or koilocytosis. Clinical follow‐up of the remaining cases with at least more years did not reveal any lower tract squamous pathologies. The diagnosis ASCUS may be due to the presence of squamous morules,^25^ or the difficulty in classifying the subtle atypia in endometrial hyperplasia. Although a diagnosis of AGC, NOS would alert clinicians to a possible upper tract pathology,^26^ a diagnosis of ASCUS may only be followed up by HPV testing and repeating cervical smears.^27^ The possibility of endometrial hyperplasia in cases HPV‐negative ASCUS should be recognized.

All cases reported as endometrial cells present in this cohort were endometrial carcinomas on follow up (Table 2). Similar findings were seen in large cohorts reviewing cervical smears reported as endometrial cells present.^28^, ^29^ Hinson et al. reported 18 endometrial pathologies in a cohort of 588 cases of endometrial cells in cervical smears, of which all were invasive carcinomas.^28^ The overall detection rate for endometrial hyperplasia on cervical smears is low, and mostly are only diagnosed as AGC but not able to be identified as neoplastic nor endometrial in origin (Table 2).

Higher FIGO staging (stage II or above), endocervical involvement, and regional nodal metastasis were found to be associated with higher detection rates. This is in line with findings reported in the literature from a meta‐analysis by Frias‐Gomez et al.^18^ and similar large scale studies,^30^, ^31^, ^32^ and also diagnostic performance of other modalities such as endometrial biopsy^33^ or metabolomics.^34^ This study further demonstrated that higher FIGO staging and endocervical involvement results in higher detection rates at consistently all cytological diagnostic categories (any glandular lesion [C3], suspicious/favor neoplastic [C4], and carcinoma [C5]), as opposed to previous studies categorizing cytologic diagnoses to positive and negative with only one binary cut‐off, most commonly by presence of any abnormality, regardless of it being squamous or glandular in nature.^31^, ^35^

**FIGURE 2:**
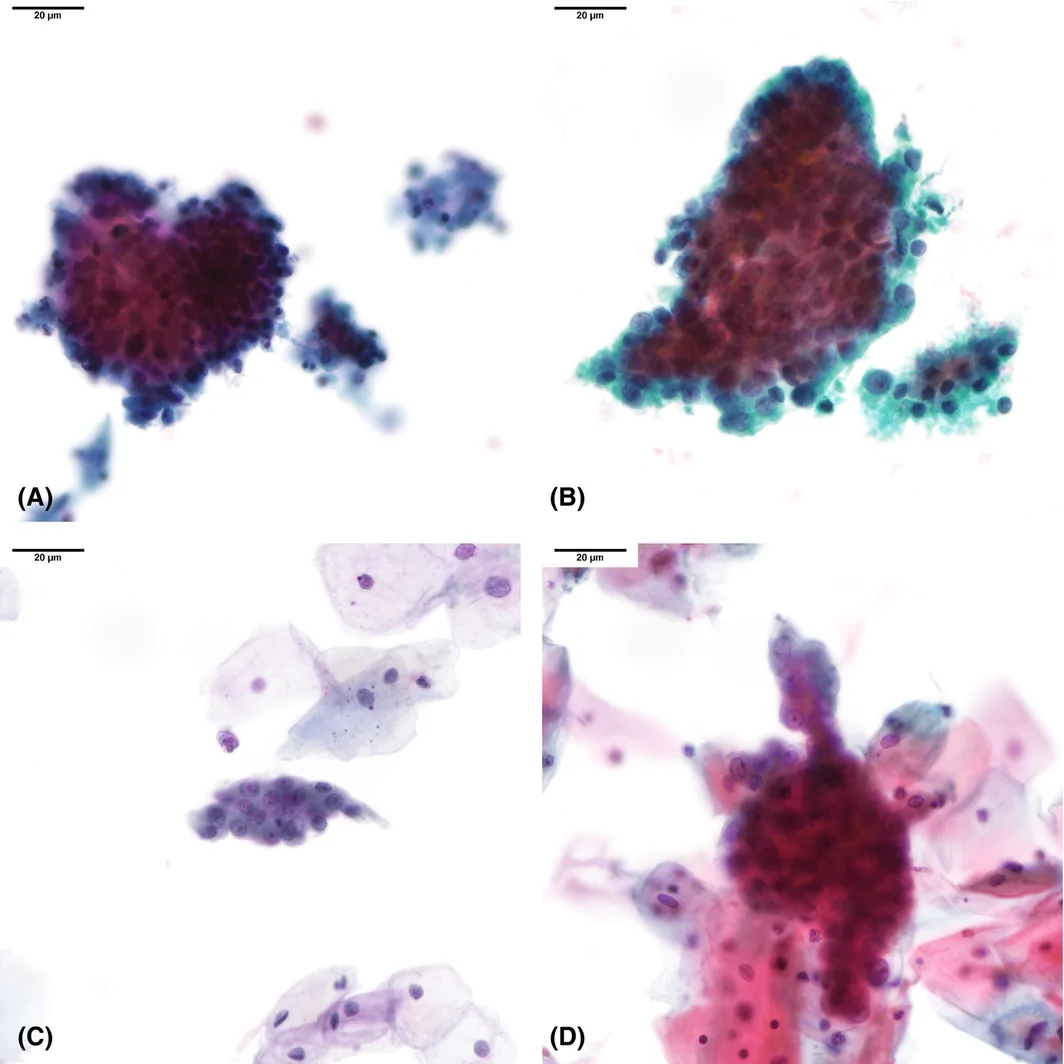
Cervical smears of endometrial carcinoma. (A) Grade II endometrial carcinoma, cytologic diagnosis was atypical glandular cells, not otherwise specified (AGC, NOS), 400× magnification; (B) Grade II endometrioid carcinoma, cytologic diagnosis was AGC, favor neoplastic, 400× magnification; (C, D) Serous carcinoma, cytologic diagnoses were adenocarcinoma, 400× magnification.

A high histological grade in endometrial carcinoma has also been associated with increased detection in cervical smears.^3^, ^36^ The meta‐analysis by Frias‐Gomez et al.^18^ seconded this association, but the definitions of positivity/abnormality were highly variable among the studies reviewed, mostly in terms of whether AGC was considered as positive or negative. There was no significant difference in detection rate between high‐grade and low‐grade endometrial carcinomas when all cytological diagnoses of glandular lesions were considered positive/abnormal, but for the categories of at least suspicious/favor neoplastic (C4) and carcinoma (C5), the detection rates for high‐grade endometrial carcinoma were significantly higher. This finding suggests that AGC, NOS should also be monitored closely as low‐grade endometrial carcinomas are not uncommonly diagnosed under the category, whereas cytologic diagnoses of higher grades may indicate high‐grade endometrial carcinomas (Figure 2).

To the best of our knowledge, this is the first comparative study exploring the performance of cervical smears on detection on endometrial hyperplasia. The detection rates for endometrial pathologies at each cytologic diagnostic category/cut‐off, as opposed to a singular/binary classification, were also reported and the effects of disease grading and staging were compared. As such, endometrial lesions, in particular endometrial hyperplasia, could be studied. However, in order to recruit a cohort of sufficient size, a lengthy retrospective collection period of more than 20 years was required, thus both conventional and liquid‐based preparations were included. A minority of cases were collected before the Bethesda system was fully implemented in our institute,^37^ although the majority could be reclassified according to the equivalent diagnostic categories, isolated cases without specification of glandular or squamous abnormality remained. The change in age cut‐off recommendation for reporting of normal endometrial cells present during the collection period of the cohort precluded meaningful statistical analysis,^38^ but the significance of normal endometrial cells on cervical smears is extensively investigated and available in published literature.^38^, ^39^

## 5 CONCLUSION

Cervical smears are useful in detection of glandular lesions of the female genital tract. The detection rates for cervical glandular were higher than endometrial lesions. Factors associated with an increased detection rate for endometrial carcinomas were a higher FIGO staging (stage II or above), presence of endocervical involvement, and regional nodal metastasis, in which FIGO grading and endocervical involvement were associated with a higher detection rate across all cytologic diagnostic categories. Histological grade was associated with increased suspicious/favor neoplastic (C4) and carcinoma (C5) diagnoses. The detection rate for endometrial hyperplasia was low, and most cases of endometrial hyperplasia were diagnosed as ASCUS or AGC, NOS, without indication of its endometrial origin. The differential diagnosis of endometrial hyperplasia in cases HPV‐negative ASCUS should be recognized, and upper tract investigations should be considered for HPV‐negative or unexplained atypical cytologic findings.

## AUTHOR CONTRIBUTIONS

**Joanna K M Ng:** Conceptualization (equal); investigation (equal); methodology (equal); writing – original draft (equal). **Bryan H. C Cheung:** Data curation (equal); investigation (equal). **Dennis H Y Lee:** Data curation (equal); investigation (equal). **Joshua J.X Li:** Conceptualization (equal); formal analysis (equal); methodology (equal); visualization (equal); writing – review and editing (equal). **Philip P. C. Ip:** Formal analysis (equal); validation (equal); writing – review and editing (equal). **Jacqueline H S Lee:** Data curation (equal); investigation (equal); writing – original draft (equal). **Carol S.Y Yeung:** Investigation (equal); resources (equal). **Mei‐Yung Yu:** Resources (equal); validation (equal).

## FUNDING INFORMATION

The authors have no funding to declare.

## CONFLICT OF INTEREST STATEMENT

The authors declare that there is no conflict of interest regarding the publication of this paper.

## ETHICAL APPROVAL

The study was approved by The Joint Chinese University of Hong Kong—New Territories East Cluster Clinical Research Ethics Committee with waiver of the requirement of written informed consent (2022.530).

## Supporting information


Table S1.

## Data Availability

The authors declare all data generated are available in the manuscript.
