# Supplementary material for: Detection rates and factors affecting thereof in endometrial hyperplasia, endometrial carcinoma, and cervical glandular lesions on cervical smear
Source: Cancer Med. 2023 Jul 27;12(17):17581–91. doi: 10.1002/cam4.6376 (PMC10523982; doi:10.1002/cam4.6376)
Supplement: Supplementary file 1 — Table S1. [file CAM4-12-17581-s001.docx]

Supplementary table 1. Histological diagnosis and pathological staging of the cohort

| Cervix |  | Hysterectomy specimens | Stage I (Tis/1A/1B)** | Stage 2 | Stage 3 (3A/3B/3C) | Stage 4 (4A/4B) |
| --- | --- | --- | --- | --- | --- | --- |
| Adenocarcinoma-in-situ | 42 |  |  |  |  |  |
| Adenocarcinoma | 58 |  |  |  |  |  |
| Squamous cell carcinoma | 164 |  |  |  |  |  |
| Carcinoma, NS | 8 |  |  |  |  |  |
|  |  |  |  |  |  |  |
| Endometrial carcinoma |  |  |  |  |  |  |
| Carcinosarcoma | 3 | 2 | 1 (0/0/1) | 0 | 0(0/0/0) | 1(1/0) |
| Clear cell carcinoma | 4 | 3 | 3 (0/2/1) | 0 | 0(0/0/0) | 0(0/0) |
| Endometrioid carcinoma |  |  |  |  |  |  |
| Grade I* | 166 | 161 | 10 (2/6/2) | 3 | 0(0/0/0) | 0(0/0) |
| Grade II | 66 | 63 | 143 (15/97/29) | 9 | 3(0/0/3) | 4(2/2) |
| Grade III | 23 | 23 | 37 (0/15/21) | 9 | 9(5/2/2) | 8(2/6) |
| Not graded | 14 | 13 | 15 (3/4/8) | 4 | 1(0/0/1) | 5(0/5) |
| Mixed carcinoma | 4 | 4 | 3 (0/0/3) | 0 | 0(0/0/0) | 1(0/1) |
| Serous carcinoma | 15 | 13 | 7 (1/5/1) | 1 | 3(0/1/2) | 2(0/2) |
| Carcinoma, NS | 17 | 11 | 6 (2/3/1) | 3 | 2(2/0/0) | 0(0/0) |
| Subtotal |  | 293 | 225 | 29 | 18 | 21 |
|  |  |  |  |  |  |  |
| Endometrial hyperplasia |  |  |  |  |  |  |
| With atypia | 117 |  |  |  |  |  |
| Without atypia | 131 |  |  |  |  |  |
|  |  |  |  |  |  |  |
| Total | 832 |  |  |  |  |  |

** Includes two cases of mucinous carcinoma diagnosed under previous versions of the World Health Organization*

*** Depth of myometrial invasion was not provided in 3 cases*

**** NS – not specified*
